# Gene model for the ortholog of *lin-28* in *Drosophila mojavensis*

**DOI:** 10.17912/micropub.biology.001096

**Published:** 2025-08-18

**Authors:** Megan E. Lawson, Jessica Cooper, Brian Schwartz, Chinmay P. Rele, Laura K. Reed

**Affiliations:** 1 The University of Alabama, Tuscaloosa, AL USA; 2 Columbus State University, Columbus, GA USA

## Abstract

Gene model for the ortholog of *lin-28* in the *Drosophila mojavensis* May 2011 (Agencourt dmoj_caf1/DmojCAF1) Genome Assembly (GenBank Accession: GCA_000005175.1 ). This ortholog was characterized as part of a developing dataset to study the evolution of the Insulin/insulin-like growth factor signaling pathway (IIS) across the genus *Drosophila* using the Genomics Education Partnership gene annotation protocol for Course-based Undergraduate Research Experiences.

**Figure 1. Genomic neighborhood and gene model for lin-28 in D. mojavensis:**
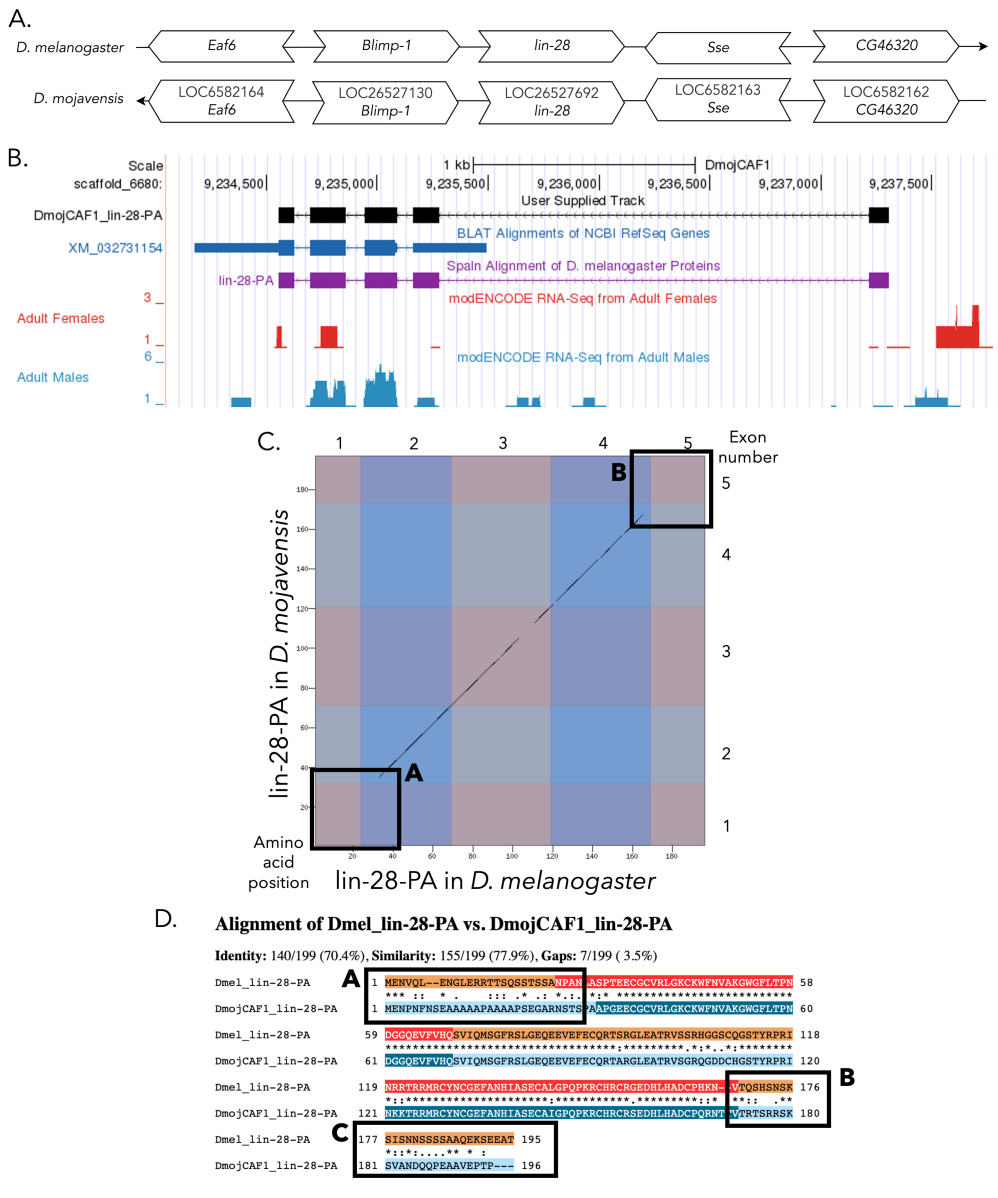
**
(A) Synteny comparison of the genomic neighborhoods for *lin-28* in *Drosophila melanogaster* and *D.* *mojavensis* .** Thin arrowheads on left and right of the image indicate the DNA strand within which the gene– *lin-28* –is located in *D. melanogaster* (top) and *D. mojavensis* (bottom). The thin arrow pointing to the right indicates that *lin-28* is on the positive (+) strand in *D.melanogaster* , and the thin arrow pointing to the left indicates that *lin-28* is on the negative (-) strand in *D. mojavensis* . The block arrows with gene names inside (wide gene arrows) pointing in the same direction as *lin-28* are on the same strand relative to the thin arrowheads, while wide gene arrows pointing in the opposite direction of *lin-28* are on the opposite strand relative to the thin underlying arrows. White gene arrows in *D. mojavensis* indicate orthology to the corresponding gene in *D. melanogaster* . Gene symbols given in the *D. mojavensis* gene arrows indicate the orthologous gene in *D. melanogaster* , while the locus identifiers are specific to *D. mojavensis* . **(B) Gene Model in GEP UCSC Track Data Hub** (Raney et al., 2014). The coding-regions of *lin-28* in *D. mojavensis* are displayed in the User Supplied Track (black); CDSs are depicted by thick rectangles and introns by thin lines with arrows indicating the direction of transcription. Subsequent evidence tracks include BLAT Alignments of NCBI RefSeq Genes (dark blue, alignment of Ref-Seq genes for *D. mojavensis* ), Spaln of D. melanogaster Proteins (purple, alignment of Ref-Seq proteins from *D. melanogaster* ), and RNA-Seq from Adult Females and Adult Males (red and light blue, respectively; alignment of Illumina RNA-Seq reads from *D. mojavensis* ; Chen et al., 2014; SRP006203 ). **(C) Dot Plot of lin-28-PA in *D. melanogaster* ( *x* -axis) vs. the orthologous peptide in *D. mojavensis* ( *y* -axis).** Amino acid number is indicated along the left and bottom; CDS (exon) number is indicated along the top and right, and CDSs are also highlighted with alternating colors. Boxes 1C-A and 1C-B highlight regions of low sequence similarity in the protein alignment. **(D) Protein alignment of lin-28-PA in *D. melanogaster* (top row) vs. the orthologous peptide in *D. mojavensis* (bottom row).** The alternating colored rectangles represent adjacent CDSs. The symbols in the match line denote the level of similarity between the aligned residues. An asterisk (*) indicates that the aligned residues are identical. A colon (:) indicates the aligned residues have highly similar chemical properties—roughly equivalent to scoring > 0.5 in the Gonnet PAM 250 matrix (Gonnet et al., 1992). A period (.) indicates that the aligned residues have weakly similar chemically properties—roughly equivalent to scoring > 0 and ≤ 0.5 in the Gonnet PAM 250 matrix. A space indicates a gap or mismatch when the aligned residues have a complete lack of similarity—roughly equivalent to scoring ≤ 0 in the Gonnet PAM 250 matrix. Box 1D-A indicates the same region of decreased sequence similarity in CDS one of lin-28-PA that is highlighted in Box 1C-A, and Boxes 1D-B and 1D-C indicate the same region of decreased sequence similarity in CDS five of lin-28-PA that is highlighted in Box 1C-B.

## Description

**Table:** 

*This article reports a predicted gene model generated by undergraduate work using a structured gene model annotation protocol defined by the Genomics Education Partnership (GEP; thegep.org) for Course-based Undergraduate Research Experience (CURE). The following information in this box may be repeated in other articles submitted by participants using the same GEP CURE protocol for annotating Drosophila species orthologs of Drosophila melanogaster genes in the insulin signaling pathway.* "In this GEP CURE protocol students use web-based tools to manually annotate genes in non-model *Drosophila* species based on orthology to genes in the well-annotated model organism fruitfly *Drosophila melanogaster* . The GEP uses web-based tools to allow undergraduates to participate in course-based research by generating manual annotations of genes in non-model species (Rele et al., 2023). Computational-based gene predictions in any organism are often improved by careful manual annotation and curation, allowing for more accurate analyses of gene and genome evolution (Mudge and Harrow 2016; Tello-Ruiz et al., 2019). These models of orthologous genes across species, such as the one presented here, then provide a reliable basis for further evolutionary genomic analyses when made available to the scientific community.” (Myers et al., 2024). “The particular gene ortholog described here was characterized as part of a developing dataset to study the evolution of the Insulin/insulin-like growth factor signaling pathway (IIS) across the genus *Drosophila* . The Insulin/insulin-like growth factor signaling pathway (IIS) is a highly conserved signaling pathway in animals and is central to mediating organismal responses to nutrients (Hietakangas and Cohen 2009; Grewal 2009).” (Myers et al., 2024). “ *D.* *mojavensis* (NCBI:txid7230) is part of the *mulleri complex* in the *repleta* species group within the subgenus *Drosophila* of the genus *Drosophila* (Wasserman 1992; Durando et al., 2000) *.* It was first described by Patterson (Patterson and Crow 1940). *D. mojavensis* specializes on rotting cactus as its host and is found in the Mojave and Sonoran Deserts of the southwestern United States and northwestern Mexico including the Baja Peninsula, as well as on the channel-islands off the coast of California (https://www.taxodros.uzh.ch, accessed 1 Feb 2023).” (Congleton et al., 2023). “ *lin-28* ( *lin-28* ) is a positive regulator of the insulin signaling (Zhu et al., 2011) and JAK-STAT (Sreejith et al., 2019) pathways. *lin-28* was discovered in *Caenorhabditis elegans* by mutations that produce heterochronic shifts in cell fate specification (Ambros and Horvitz 1984). Homologs were identified later in other animals, including *Drosophila* , *Xenopus* , mouse, and human (Moss and Tang 2003). lin-28 binds to many mRNA molecules to regulate their translation or stability (Balzer and Moss 2007; Cho et al., 2012). In *Drosophila* , lin-28 binds to the *insulin-like receptor (InR)* mRNA and stimulates the symmetric division of intestinal stem cells in response to nutrients (Chen et al., 2015; Luhur and Sokol 2015). In mammals, LIN28, in combination with OCT4, SOX2, and NANOG, can reprogram differentiated somatic cells to pluripotency (Yu et al., 2007).” (Lawson et al, 2025)

We propose a gene model for the *D. mojavensis* ortholog of the *D. melanogaster* *lin-28* gene. The genomic region of the ortholog corresponds to the uncharacterized protein LOC26527692 (RefSeq accession XP_032587045.2 ) in the May 2011 (Agencourt dmoj_caf1/DmojCAF1) Genome Assembly of *D. mojavensis* (GenBank Accession: GCA_000005175.1 ; Drosophila 12 Genomes Consortium et al., 2007). This model is based on RNA-Seq data from *D. mojavensis* ( SRP006203 - Chen et al., 2014) and *lin-28* in *D. melanogaster* using FlyBase release FB2023_02 ( GCA_000001215.4 ; Larkin et al., 2021; Gramates et al., 2022; Jenkins et al., 2022).

***Synteny***

The reference gene, *lin-28 ,* occurs on chromosome 3L in *D. melanogaster* and is flanked upstream by *Blimp-1* and *Esa1-associated factor 6* ( *Eaf6* ) and downstream by *Separase* ( *Sse* ) and *CG46320 .* The *tblastn* search of *D. melanogaster* lin-28-PA (query) against the *D. mojavensis* (GenBank Accession: GCA_000005175.1 ) Genome Assembly (database) placed the putative ortholog of *lin-28* within scaffold_6680 ( CH933809.1 ) at locus LOC26527692 ( XP_032587045.2 )— with an E-value of 2e-36 and a percent identity of 40.41%. Furthermore, the putative ortholog is flanked upstream by LOC26527130 ( XP_032587044.1 ) and LOC6582164 ( XP_002007868.1 ), which correspond to *Blimp-1* and *Eaf6* in *D. melanogaster* (E-value: 0.0 and 2e-123; identity: 76.07% and 85.02%, respectively, as determined by *blastp* ; Figure 1A, Altschul et al., 1990). The putative ortholog of *lin-28* is flanked downstream by LOC6582163 ( XP_032587039.1 ) and LOC6582162 ( XP_032587042.1 ), which correspond to *Sse* and *CG46320* in *D. melanogaster* (E-value: 0.0 and 5e-32; identity: 60.31% and 92.59%, respectively, as determined by *blastp* ). The putative ortholog assignment for *lin-28* in *D. mojavensis* is supported by the synteny of the genomic neighborhood being completely conserved across both species, and all *BLAST* results used to determine orthology indicate high-quality matches.

***Protein Model***

*lin-28* in *D. mojavensis* has one protein-coding isoform, lin-28-PA ( Figure 1B ). mRNA isoform *lin-28-RA* contains five CDSs (coding exons). Relative to the ortholog in *D. melanogaster* , the RNA CDS number is conserved, as *lin-28* in *D. melanogaster* also has only one isoform with five CDSs. The sequence of lin-28-PA in *D. mojavensis* has 70.4% identity with the protein-coding isoform lin-28-PA in *D. melanogaster* , as determined by *blastp* ( Figure 1C ). Coordinates of this curated gene model are stored by NCBI at GenBank/BankIt (accession **BK065267** ). These data are also archived in the CaltechDATA repository (see “Extended Data” section below).

***Special characteristics of the protein model***

**Regions lacking sequence similarity in the first and last CDS of *lin-28-RA***

Boxes 1C-A, 1C-B, 1D-A, and 1D-B highlight regions of decreased sequence similarity in the alignment of the first CDS of *lin-28-RA* in *D. melanogaster* and *D. mojavensis.* These regions are found primarily within the first and last CDSs of the gene, which both have decreased sequence similarity compared to the rest of the protein alignment. However, all other CDSs of *lin-28-RA* show very high sequence conservation, which in combination with the complete conservation of synteny indicates that this is still likely the correct ortholog assignment for *lin-28* in *D. mojavensis.*

## Methods

Detailed methods including algorithms, database versions, and citations for the complete annotation process can be found in Rele et al. (2023). Briefly, students use the GEP instance of the UCSC Genome Browser v.435 (https://gander.wustl.edu; Kent WJ et al., 2002; Navarro Gonzalez et al., 2021) to examine the genomic neighborhood of their reference IIS gene in the *D. melanogaster* genome assembly (Aug. 2014; BDGP Release 6 + ISO1 MT/dm6). Students then retrieve the protein sequence for the *D. melanogaster* reference gene for a given isoform and run it using *tblastn* against their target *Drosophila* species genome assembly on the NCBI BLAST server (https://blast.ncbi.nlm.nih.gov/Blast.cgi; Altschul et al., 1990) to identify potential orthologs. To validate the potential ortholog, students compare the local genomic neighborhood of their potential ortholog with the genomic neighborhood of their reference gene in *D. melanogaster* . This local synteny analysis includes at minimum the two upstream and downstream genes relative to their putative ortholog. They also explore other sets of genomic evidence using multiple alignment tracks in the Genome Browser, including BLAT alignments of RefSeq Genes, Spaln alignment of *D. melanogaster* proteins, multiple gene prediction tracks (e.g., GeMoMa, Geneid, Augustus), and modENCODE RNA-Seq from the target species. Detailed explanation of how these lines of genomic evidenced are leveraged by students in gene model development are described in Rele et al. (2023). Genomic structure information (e.g., CDSs, intron-exon number and boundaries, number of isoforms) for the *D. melanogaster* reference gene is retrieved through the Gene Record Finder (https://gander.wustl.edu/~wilson/dmelgenerecord/index.html; Rele et al *.,* 2023). Approximate splice sites within the target gene are determined using *tblastn* using the CDSs from the *D. melanogaste* r reference gene. Coordinates of CDSs are then refined by examining aligned modENCODE RNA-Seq data, and by applying paradigms of molecular biology such as identifying canonical splice site sequences and ensuring the maintenance of an open reading frame across hypothesized splice sites. Students then confirm the biological validity of their target gene model using the Gene Model Checker (https://gander.wustl.edu/~wilson/dmelgenerecord/index.html; Rele et al., 2023), which compares the structure and translated sequence from their hypothesized target gene model against the *D. melanogaster* reference gene model. At least two independent models for a gene are generated by students under mentorship of their faculty course instructors. Those models are then reconciled by a third independent researcher mentored by the project leaders to produce the final model. Note: comparison of 5' and 3' UTR sequence information is not included in this GEP CURE protocol (Gruys et al., 2025).

## Data Availability

Description: A GFF, FASTA, and PEP of the model. Resource Type: Model. DOI: https://doi.org/10.22002/rxg68-9gt23
